# Gliadin and glutenin genomes and their effects on the technological aspect of wheat-based products

**DOI:** 10.1016/j.crfs.2023.100622

**Published:** 2023-10-28

**Authors:** Kiana Pourmohammadi, Elahe Abedi, Seyed Mohammad Bagher Hashemi

**Affiliations:** Department of Food Science and Technology, Faculty of Agriculture, Fasa University, Fasa, Iran

**Keywords:** Genetic characteristics, Genome editing, Bread making quality, Glutenin genomes, Gliadin genomes

## Abstract

Wheat is the most important crops worldwide, providing about one-fifth of the daily protein and calories for human consumption. The quality of cereal-based products is principally governed by the genetic basis of gluten (glutenin and gliadin proteins), which exists in a wide range of variable alleles and is controlled by clusters of genes. There are certain limitations associated with gluten characteristics, which can be genetically manipulated. The present review aimed to investigate the correlation between the genetic characteristics of gluten protein components and wheat-based product's quality. According to various references, *Glu-B1d (6 + 8)*, *Glu-B1h (14 + 15)* and *Glu-B1b (7 + 8)* are related to higher gluten strength and pasta quality, while, subunits Dx2 + Dy12 and Dx5 + Dy10, are usually present at the Glu-D1 locus in bread wheat, resulted in lower cooked firmness in pasta. Moreover, introducing Gli-D1/Glu-D3 and Glu-D1 loci into durum wheat genomes, causing to provide the maximum values of gluten index in pasta products. 1Dx5 + 1Dy10 alleles determine the level of increase in dough's consistency, elasticity, viscosity, and extensibility quality of baking and appropriate bread loaf volume, while 1Dx2 + 1Dy12 as the alleles associated with poor baking quality, being more suitable for soft wheat/pastry end uses. Bx7, Bx7^OE^, 1Bx17 + 1By18, 1Bx13 + 1By16, Bx7 + By9 and 1Bx7 + 1By8 at Glu-B1*alleles and* 1Ax2* found on Glu-A1, augmented dough strength and has positive effects on consistency, extensibility, viscosity, and elasticity of bread dough. Breeding programs by genome editing have made gluten a promoting component for improving cereal-based products.

## Highlights

• Improving the quality of cereal-based products by genome editing.
• Efficiency of alleles՚ silencing or expression in gliadin and glutenin on bread quality.
• Genetic modification is eco-friendly effective manipulation for wheat breeding.

## Graphical abstract

**Figure:**
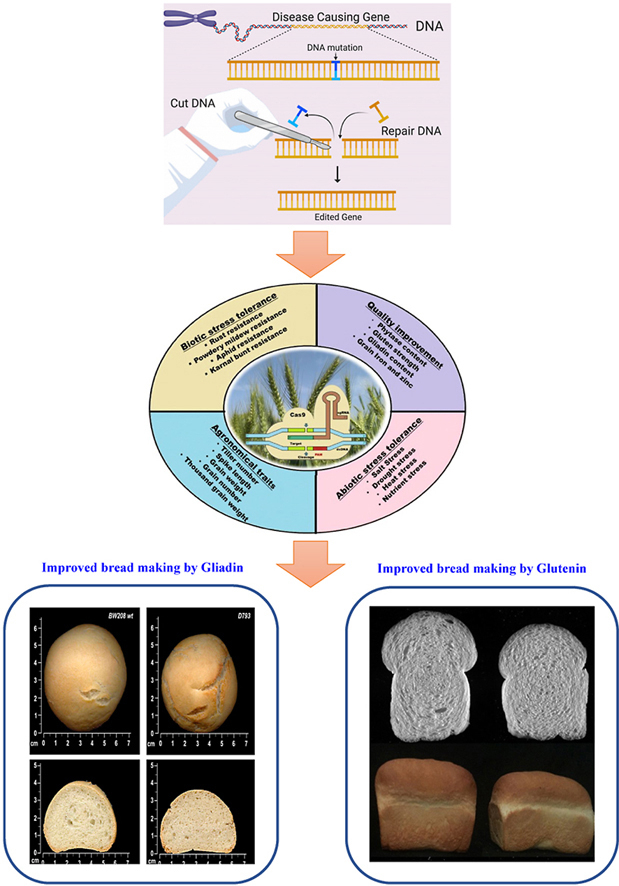

## 1 Introduction

The primary staple crop, cultivated to be processed into different food products, is known to be wheat (*Triticum aestivum* L., AABBDD). As some examples of these food items, we could mention noodles, macaroni, bread, pasta, spaghetti, cakes, biscuits, pizzas, and chapatti (Yi Li et al., 2020; Patil et al., 2011). Protein accounts for 8–16% of mature wheat grains while total flour protein comprises gluten proteins by up to 80–85%; this is believed to be the main element when assessing the quality of baked products and the texture of processed food (E Abedi and Pourmohammadi, 2020a, Abedi and Pourmohammadi, 2020b; Abedi et al., 2018; Abedi and Pourmohammadi, 2021; Pourmohammadi and Abedi, 2021a, 2021b). Gluten, a complex mixture of proteins found in wheat, plays a crucial role in determining the viscoelastic properties of dough and the quality of bread. Gluten proteins are usually classified in two main groups, namely gliadins and glutenins. Gliadins influence the extensibility and viscous nature while glutenins play a role in dough's elasticity and strength (Abedi and Pourmohammadi, 2020a, Abedi and Pourmohammadi, 2020b, 2021; Abedi et al., 2018; Majzoobi and Abedi, 2014; Majzoobi et al., 2012; Shewry, 2019; Wang et al., 2020). In grains, the decline in glutenins has been reported to be compensated for by the rise in gliadin content; this highlights the proper system of balancing gluten proteins in wheat (Pistón et al., 2011; van den Broeck et al., 2009). Nevertheless, there is currently a variety of wheat with different features, which is known to be the result of wheat adaptability to various eco-climatic conditions and the fact that it has been deliberately bred for some particular traits (Yi Li et al., 2020).

However, the composition and properties of gluten can vary significantly among different wheat varieties and cultivars. Some limitations associated with gluten characteristics include:

1. Gluten strength and elasticity: The strength and elasticity of gluten are important factors in bread-making. Gluten with low strength and elasticity can result in poor dough handling properties, leading to bread with reduced volume and a dense texture. Genetic manipulation aimed at enhancing the strength and elasticity of gluten could potentially improve bread quality.
2. Gluten sensitivity and allergenicity: Gluten proteins contain certain components (gliadins and glutenins) that can trigger adverse reactions in individuals with gluten sensitivity or celiac disease. Genetic manipulation could potentially reduce or eliminate the presence of specific gluten components responsible for these adverse reactions, allowing individuals with gluten sensitivity to consume bread without experiencing negative health effects.
3. Dough rheology: The rheological properties of dough, such as extensibility and elasticity, are influenced by the gluten protein composition. Genetic manipulation could target specific gluten protein components to optimize dough rheology, resulting in improved bread-making performance and quality.
4. Nutritional composition: Gluten proteins contribute to the nutritional composition of bread. However, some individuals may have dietary restrictions or preferences that require specific modifications to the gluten protein composition. Genetic manipulation could be utilized to tailor the nutritional profile of gluten proteins to meet specific dietary needs or preferences.

**Fig. 1:**
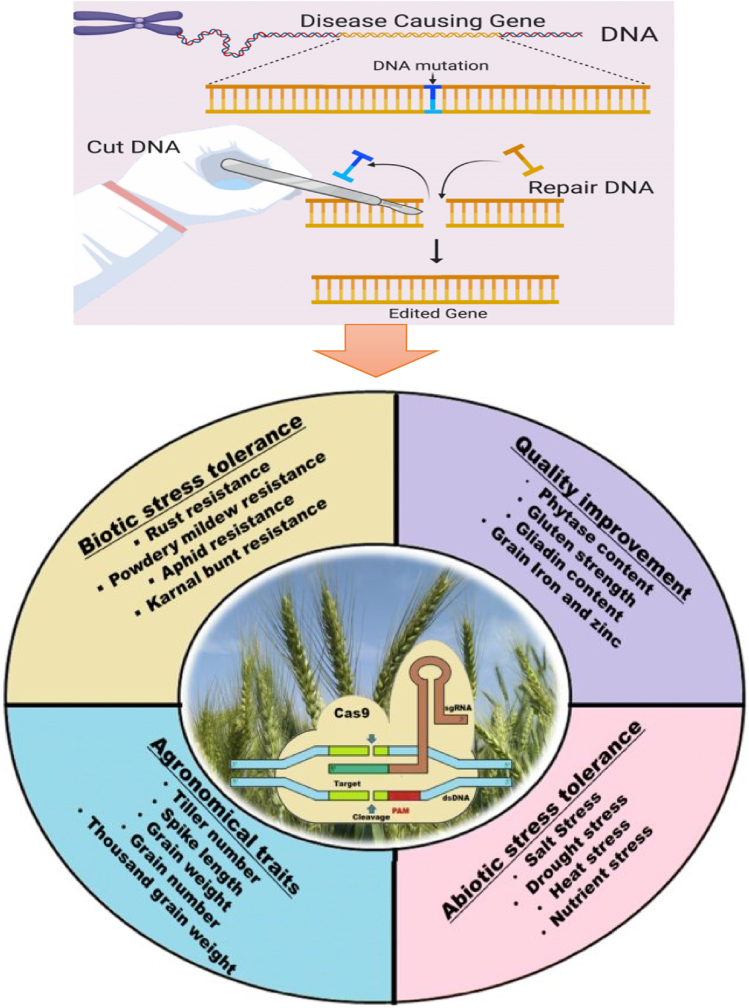
The importance of genome editing in cereal-based products.

Improvement in wheat baking quality is of paramount importance for the programs aiming at plant breeding. These programs could help introduce wheat varieties broadly accepted and applied by farmers. Thus, high-quality grain will be produced, resulting in a desirable end product. On the other hand, the genetic aspects of gluten have a crucial role in dough rheology and end-use properties (Fig. 1); therefore, genetic manipulation of gluten would lead to a higher-quality product as reported by several researchers (Chen et al., 2021; Gao et al., 2018a; Liu et al., 2016a; Song et al., 2020). Breeding programs concerning gluten allelic variations have attracted a great deal of scientific attention over the past decades since they could improve bread-making quality considerably. In this regard, numerous studies have revealed that genome editing has made gluten a promoting component for bread-making quality (Cho et al., 2018; Gao et al., 2018b; Hernández-Estrada et al., 2017; Kaur et al., 2014; Yiwen Li et al., 2015; Liu et al., 2016b; Song et al., 2020; D. Wang et al., 2018). By investigating the genetic characteristics of gluten protein components and their correlation with bread-making quality, researchers can identify specific genes or genetic markers associated with desirable bread-making traits. This knowledge could be used to develop wheat varieties with improved gluten characteristics, leading to enhanced bread quality and meeting the diverse needs of consumers. It is worth noting that genetic manipulation should be approached with caution, considering potential ethical, safety, and regulatory considerations. Any genetic modifications should be thoroughly evaluated for their impact on human health, environmental sustainability, and consumer acceptance. This study was conducted to present a comprehensive overview of the genetic characteristics associated with the components of gluten protein, the genomes affecting dough and bread quality, and the genetic modifications that could have improved the quality of cereal-based products so far.

## 2 Genetic characteristics of gliadin and gliadin genomes affecting bread making quality

**Fig. 2:**
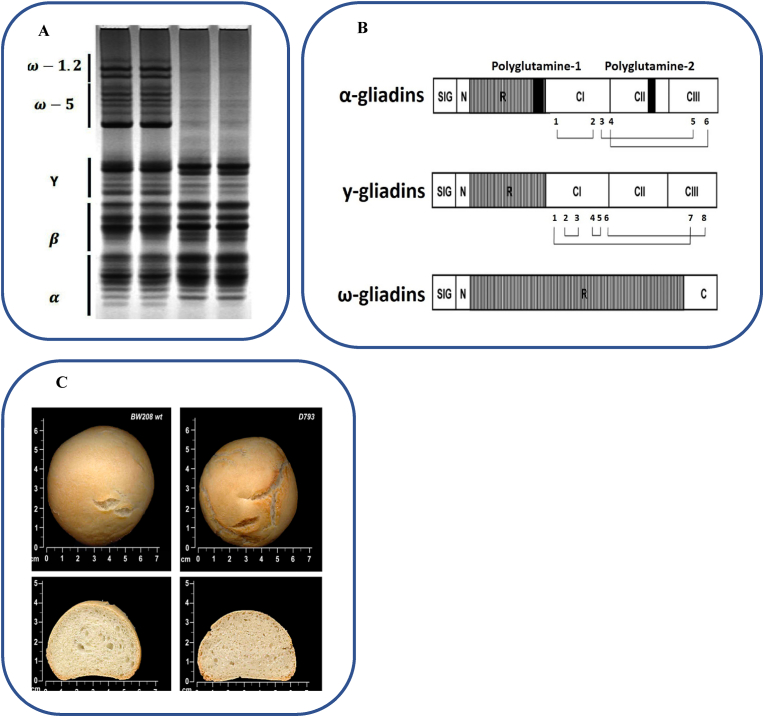
A) Schematic structure of α, γ and ω gliadin proteins; B) Wheat gliadin proteins of control (3xC) and ω -gliadin free (3xN) lines separated by A-PAGE (Waga and Skoczowski, 2014); C) Loaves and slices of wild-type BW208 and reduced-gliadin line D793 (Gil-Humanes et al., 2014).

As an alcohol-soluble storage protein, gliadin makes up about 40–50% of total flour proteins even though total gliadins distribution majorly depend on genotype-related and environmental elements (Fig. 2). Gliadin is a combination of monomeric proteins which result in extensibility and viscosity of the wheat dough (Wang et al., 2020; Žilić, 2013). Polyacrylamide gel electrophoresis has been demonstrated to include four main categories, namely α- (25–35 kDa), β- (30–35 kDa), γ- (35–40 kDa), and ω- (55–75 kDa) gliadins (Békés et al., 2017; Metakovsky et al., 2018). According to another classification and the analysis based on primary structure and molecular weights, gliadins classify into ω5-, ω1, 2-, α/β-, and γ -gliadins. Proportional to ω-gliadins, α- and γ-gliadins are more abundant despite the heavy dependence of total gliadins distribution on genotype-related and environmental factors (Žilić, 2013). Furthermore, in the make-up of amino acid, ω-gliadin, defined as S-poor (ω-) gliadins, is different from α- and γ-gliadins known as S-rich gliadin subunits (Fig. 2A). Gliadin is encoded by multigene families (Dubois et al., 2016; Metakovsky et al., 2018; Shewry, 2019). Based on various studies in the field of chromosomal location, the genes that encode gliadin could be seen on the chromosomes of homoeologous group 1 (Gli-A1, -B1 and -D1 loci) and group 6 (Gli-A2, -B2 and -D2 loci) short arm (Fig. 2B). Research on genetic crossbreeding has indicated that clusters of genes, *Gli-A1*, *Gli-Bl*, and *Gli-Dl*, take control of γ- and ω-gliadins. These clusters are seen on group 1 chromosomes short arms. Moreover, *Gli-A2*, *Gli-B2*, and *Gli-D2*, which are located on group 2 chromosomes short arms, control α- and β-gliadins. In addition, a number of minor gliadin loci are seen on 1AS (Gli-A3, -A5 and -A6), 1BS (Gli-B3 and -B5), as well as 1DS (Gli-D4 and -D6) (Fig. 2C) (Balakireva and Zamyatnin Jr, 2016; Ozuna et al., 2015; Urade et al., 2018; Utebayev et al., 2019).

### 2.1 α -gliadins

Constituting between 15% and 30% of the total seed storage proteins in wheat, α-gliadins are believed to be of great importance (Altenbach et al., 2014). Gli-2 locus encodes α-type gliadins of hexaploid *Triticum aestivum*. Gli-2 locus is located on the short arm of group 6 chromosomes. Estimation of α-gliadin copy number was reported to be in the range between 25 and 35 copies up to 100 or even 150 copies per haploid genome, making α-gliadin gene family the most complex protein (Fig. 2). In terms of evolution, *Gli-2* loci, which encode α-gliadins, are known as the newest genomic region among the three main regions that harbor prolamin genes of wheat. That is because the closely linked species in *Triticeae* tribe, such as rye and barley, do not carry α-gliadin genes (Huo et al., 2019). Various studies have shown the impact of gliadin on dough and bread quality; for instance, Noma et al. (2019 and 2023) reported the characteristics of α-gliadins in Japanese wheat cultivars to be able to improve end-use quality (Noma et al., 2019, 2023). A number of papers have positively associated α/β and γ-gliadins with loaf volume and development time (Fig. 2). In this regard, adding growing levels of gliadin to flour reduced dough strength overall with the following order: ω1 > γ > β > α-gliadins; this causes increased elastic modulus values owing to raised concentrations found in uncrossed-linked material in comparison to native gluten. Additionally, in different gliadins, the rising order of slopes was reported as follows: β > γ > α = ω 1> ω 2; this suggests that glutens with ω- and α-gliadins are rather less crossed-linked compared to those with β- and γ-gliadins. The quality of dough has been also reported to decline with the rise in ω-gliadins proportion and the fall in α/β- and γ-gliadins (Gil-Humanes et al., 2012). According to Van den Broeck et al. (2009), technological properties decline significantly by deletions of the α-gliadin locus situated on chromosome 6D short arm. That is while these properties remain unchanged with deletions in chromosome 1D short arm (ω-gliadin, γ-gliadin, and LMW loci) (van den Broeck et al., 2009). In another study by Van den Broeck et al. (2011), bread-making quality was positively affected by deletion of Gli-D2 locus located on chromosome 6D short arm, containing the α-gliadin genes (van den Broeck et al., 2011). Moreover, another study showed increased dough strength, despite slightly reduced loaf volume, due to α-gliadins silencing in bread wheat, contributing to a 63% decline in α-gliadin content.

### 2.2 γ-gliadins

In wheat gluten family, the most important members are known to be γ-gliadins. In GenBank, according to sequence information concerning γ-gliadin genes from different wheat and several species, there are 34 complete or nearly complete open reading frames as well as 66 partial sequences, where γ-gliadin genes are considered to be a 10- to 40-member multigene family in wheat (Qi et al., 2009; D.-W. Wang et al., 2017). These genes were found to be encoded by the *Gli-1* loci on the short arm of homologous group 1 chromosome (Barak et al., 2015). γ-gliadins protein sequences normally commence with a signal peptide. Subsequently, these sequences continue in the following order: N-terminal non-repetitive domain, a highly variable repetitive domain, a non-repetitive domain (with six conserved cysteine residues), a rich glutamine domain, as well as the C-terminal non-repetitive domain (with two conserved cysteine residues) (Zhu et al., 2021). Dough rheology, in addition to its end-use properties, are highly affected by the accumulation of γ-gliadins (Ma et al., 2019; D. Wang et al., 2020). Numerous papers have indicated γ-gliadins to be negative regulators of wheat quality (Zhu et al., 2021).

Adding γ-gliadin to wheat flour decreases the time needed for mixing along with its resistance against extension. It also reduces dough's gluten strength (Zhu et al., 2021). Regarding durum wheat, it was previously shown that γ-42 gliadin is related to the decline in SDS sedimentation volume, implying decreased protein quality. In support of this, Gil-Humanes et al. (2014) demonstrated that in wheat, γ-gliadins silencing through RNAi results in a 33%–43% fall in the content of γ-gliadin, on top of a rise in SDS sedimentation volume (Gil-Humanes et al., 2012). Hasrak et al. (2019) also showed the importance of γ-gliadin in bread quality (Hasrak et al., 2019). In contrast, based on the results reported by Pistón et al. (2011), γ-gliadins do not essentially or functionally affect the quality of breadmaking (Pistón et al., 2011). They also reported that other gluten proteins could function as an alternative to these genes. Since γ-gliadin is controlled by *Gli-A1*, *Gli-Bl*, and *Gli-Dl*, Sherman et al. (2018) reported allelic variation at the *Gli-B1* locus to have a significant effect on dough characteristics and the quality of bread production, regardless of its genetic backgrounds or the environmental conditions (Sherman et al., 2018). TaGli-γ-2.1 has been considered as a subgroup of γ-gliadin multigene family. It has been reported to be expressed preferentially in the later period of grain filling. According to Zhou et al. (2022), dough stability time is significantly reduced by adding TaGli-γ-2.1 protein fragment to strong gluten wheat flour (Zhou et al., 2022).

### 2.3 ω-gliadins

Total protein of flour is constituted by ω-gliadins by up to 5%–10%. This percentage depends on the plant's growth conditions and cultivar. Moreover, there are repetitive sequences in ω-gliadins, containing big amounts of glutamine and proline (∼ 68–73%), but lacking cysteine (Altenbach et al., 2020). The proteins are divided in two groups, namely ω-5 and ω-1,2 gliadins, differing in N-terminal sequences. The encoding of ω-5 gliadins happens at *Gli-1* locus on chromosome 1B short arm in hexaploid wheat; meanwhile, that of ω-1,2 gliadins is seen on chromosomes 1A and 1D (Tye-Din et al., 2010). Various studies have revealed that ω-gliadins editing would affect dough and bread quality (Fig. 2). In this regard, Waga and Skoczowski (2014) exhibited higher quality, remarkably higher strength of dough, as well as decreased extensibility of dough in progenies containing inactive genes at the *Gli-D1* locus (Waga and Skoczowski, 2014). An elevated HMW polymeric proteins to monomeric gliadins ratio is believed to be the most likely reason behind higher quality in the absence of ω gliadins (Waga and Skoczowski, 2014). Neither flour functionality nor the expression of other grain proteins was proved to be affected by ω-5 gliadins removal from wheat. Contrarily, removing these genes bettered dough properties and augmented stability of proteins; this suggests ω-5 gliadins' negative impact on the quality of flour. Similarly, Altenbach et al. (2020) stated that eliminating ω-5-gliadins positively affected the end-use quality of flour (Altenbach et al., 2020). In addition, according to Altenbach et al. (2019), there was an improvement in both tolerance and mixing time once ω −1,2 gliadins were absent. Therefore, silencing ω-5- and ω −1,2 gliadins would improve the technological aspects of wheat (Altenbach et al., 2019).

## 3 Genetic characteristics of glutenin and glutenin genomes affecting bread making quality

### 3.1 HMW-GS

**Fig. 3:**
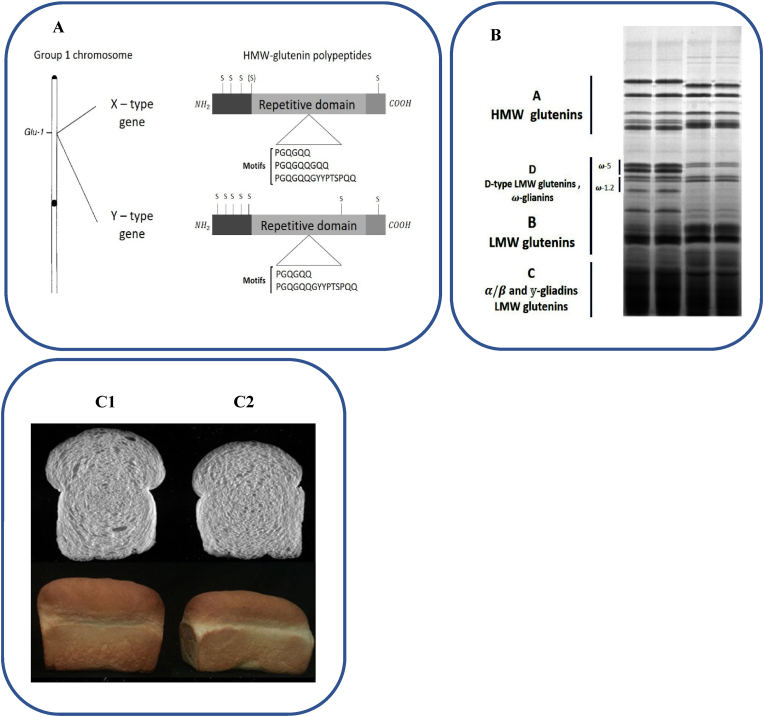
A) Electrophoretic pattern of HMW, LMW and gliadin proteins. B) Schematic diagram of the gene loci of a high-molecular-weight glutenin subunit (HMW-GS) in wheat chromosome 1: the genes coding the synthesis of HMW-GS are located on the long arms of group 1 chromosomes 1A, 1B, and 1D (Li et al., 2020). C) Loaf bread made from HMW-overexpression (C1) and native (C2) flour.

**Fig. 4:**
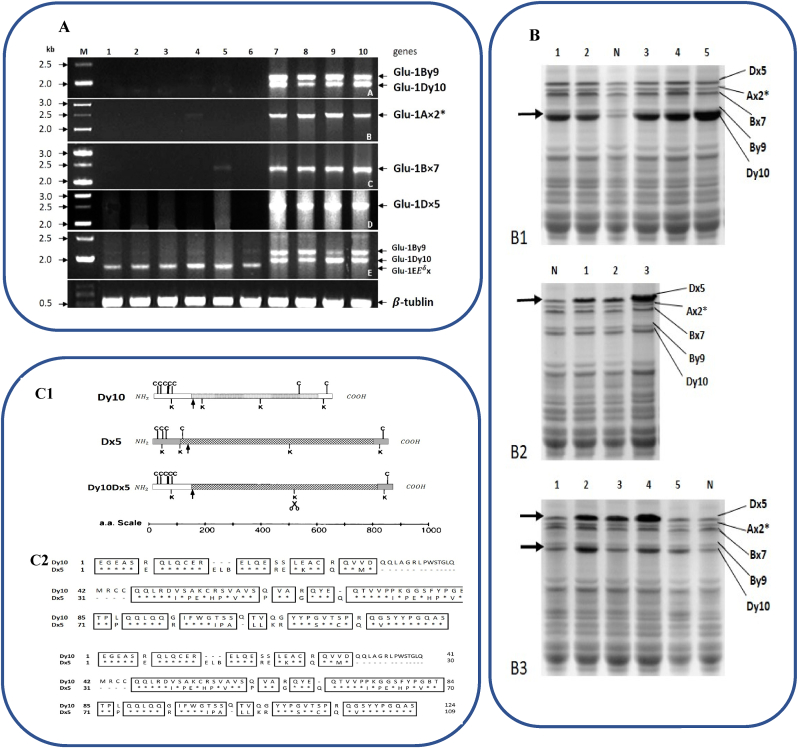
(A) Expression analysis of HMW-GS genes (Glu-1By9, Glu-1Dy10, Glu-1Ax2*, Glu-1Bx7, Glu-1Dx5, Glu-1By9, Glu-1By9, Glu-1Ebx and β-tubulin) using RT-PCR. Almost all the HMW-GS genes are silenced in transgenic line LH-11 and M, marker; 1–6 cDNA from the seeds of transgenic line LH-11. The numbers on the left side of the figure indicate the sizes (kb) of the PCR bands (Zhang et al., 2018). B) SDS-PAGE of seed protein extracts from homozygous transgenic wheats (numbered lanes) and their non-transformed parent (N). The positions of the five HMW-GS are indicated. Arrows indicate the locations of the HMW-GS increased in the transformants. (B1) Seed protein extracts from transgenic wheat with increases only in Dy10. (B2) Seed protein extracts from transgenic wheats with increases only in Dx5. (B3) Seed protein extracts from transgenic wheats with increases in both Dx5 and Dy10 (Blechl et al., 2007). C1) Schematic diagram of subunits Dy10, Dx5, and the recombinant Dy10-Dx5 polypeptide. C2) amino acid sequence alignment of the N-terminal domains of the mature Dy10 and Dx5 subunits. Identical amino acids are asterisked and boxed, similar amino acids are boxed, gaps are dashed, and cysteine residues are in bold type.

As a storage protein, glutenin is an in-soluble protein, partially soluble in dilute acid or alkali solutions. Glutenin is a complex combination of polymers linked with disulfide bonds, containing high molecular weight glutenin subunits (HMW-GSs, MW of 67,000–90,000 Da) as well as those with a low molecular weight (LMW-GSs, MW of 30,000–45,000 Da) (Fig. 3A and B; Table 2). Concerning wheat end-use quality, HMW-GSs are known as the key determinants. Regarding the genetic aspect in hexaploid aestivum wheat, there are six HMW-GS genes on homologous chromosome 1A, 1B, and 1D (Glu-A1, Glu-B1, and Glu-D1) long arm; however, there are four HMW-GS genes seen on 1A and 1B (Glu-A1, Glu-B1) in tetraploid durum wheat (Table 1) (Yi Li et al., 2020). Research on alleles frequency at each of the loci (A1, B1, D1) have implied the existence of at least three alleles at *Glu-A1* while at *Glu-B1*, 11 alleles and at *Glu-D1*, six alleles have been documented. These results were obtained by isolating HMW-GSs from SDS-PAGE (Fig. 3, Fig. 4; Table 2) (Yi Li et al., 2020). The genes coding for 1Bx, 1Dx and 1Dy subunits are consistently expressed whereas those coding for 1Ax and 1By subunits are only expressed in certain cultivars. Additionally, at the outset of the repetitive domain, subunit 1Dx5 includes an additional cysteine; meanwhile, subunits 1Bx14 and 1Bx20 have only two cysteine residues, one of which is located in the N-terminal region while the other one is situated in the C-terminal domain. It was also reported that subunit 1Ay is sometimes present in hexaploid wheat (Roy et al., 2020), but more frequently seen in A-genome diploids (D. Jiang et al., 2009). Regarding the differences between genomes, Li et al. (2020) depicted subloci-related differences to be more significant than those of homoeoalleles (J. Li et al., 2023). Accordingly, the differences found between Glu-D1x (encoding x-type subunits) and Glu-D1y genes (encoding y-type subunits) were found to be more significant than those between Glu-D1y and Glu-B1y genes (Yi Li et al., 2020). There are two closely linked HMW-GS genes at each locus, one of which is “x” type while the other is “y” type, according to their electrophoretic mobility with relative molecular masses respectively ranging between 82,000 and 90,000 Da and 60,000 and 80,000 Da (Peng et al., 2015). There is a typical three-domain structure in both x-type (larger subunit) and y-type (smaller subunit) of HMW-GS, including N-terminal cysteines in y-type and single C-terminal cysteine in x-type subunits (Lafiandra and Shewry, 2022). Generally, the majority of x-type subunits have four cysteines, with three of them being in N-terminal and one of them in C-terminal domains. y-type subunit however contains seven cysteines: five in N-terminal domains, one in the central repetitive domain, as well as one in the C-terminal domain. Therefore, to improve the quality of baking, y-type subunits are of greater importance owing to their enhanced capability of generating inter- and intra-chain disulfide bonds (Fig. 3A) (Yiwen Li et al., 2015; Peng et al., 2015). Various studies have investigated the positive and negative effects of different genomes on dough and bread quality. Overall, based on the alleles studied in different papers, *Glu-D1d* allele-containing wheat, where 1Dx5 and 1Dy10 are encoded, has indicated the highest ability to improve the quality of bread making. Nevertheless, the lowest scores in this regard belong to *Glu-A1c* (null), *Glu-B1a* (subunit 7), *Glu-B1d* (subunit 6 + 8), and *Glu-D1c* (subunit 7 + 9), all of which negatively affect bread making quality (Fig. 3 A and B) (Yi Li et al., 2020). Moreover, the quality of bread making has been found to be positively affected by the alleles that encode 1Ax1, 1Ax2*, 1Bx7 +1By9, 1Bx14 + 1By15, 1Bx17 + 1By18, and 1Dx5 + 1Dy10 subunits (Alemu et al., 2021; Guzmán et al., 2022; P. Jiang et al., 2019). According to the study by Guzmán et al. (2022), among various glutenin alleles, the followings are attributed to higher strength of gluten, its good extensibility, and higher volume of bread loaf: *Glu-A1a* (subunit 1); *Glu-A1b* (subunit 2*); *Glu-B1al* (subunits 7^OE^+8); *Glu-B1i* (subunits 17 + 18); *Glu-B1f* (13 + 16); *Glu-D1d* (subunits 5 + 10); *Glu-A3b* (subunits 5); *Glu-A3d* (subunits 6 + 11); *Glu-A3f*, *Glu-B3c*; *Glu-B3d*. On the other hand, an overall low-quality profile has been pertained to the following alleles: *Glu-A1c* (Null); *Glu-B1a* (subunit 7); *Glu-B1d* (subunits 6 + 8); *Glu-D1a* (subunits 2 + 12); *Glu-A3e* (subunit 11); *Glu-B3j* (Guzmán et al., 2022). Meanwhile, studying the deletion of HMW-GS loci combinations showed that the role of each HMW-GS contributing to dough processing characteristics could be follow this order: 1Dx5 + 1Dy10 > 1Bx17 + 1By18 > 1Ax1 + Null (P. Jiang et al., 2019). Numerous works have however argued that all HMW-GSs are conducive to augmenting the quality of dough, thereby bread processing, yet there are differences among them in terms of magnitude; that is due to the absence of HMW-GS with less effect, like 1Dx2, 1Dy12, 1Bx20, and 1By20, resulting in low-quality flour processing in wheat mutants (H. Chen et al., 2021; Gao et al., 2018a; Liu et al., 2016a; Song et al., 2020; D. Wang et al., 2020) (Fig. 3). Furthermore, several studies have revealed that the number of cysteine residues (additional or less) plays a crucial role in the formation of glutenin polymers, thereby dough and bread quality; for instance, in Bx17, serine, serine, and glutamine replace cysteine, proline, and arginine in Dx5; accordingly, Dx5 has additional cysteine, which might form another interchain bond, facilitating the superior bread properties (Yiwen Li et al., 2015; Lutz et al., 2012). As for HMW-GSs with less cysteine residues, subunits 1Bx20 and 1Bx14*, having two cysteine residues in comparison with subunit 1Bx7 containing four cysteine residues exhibited less glutenin polymer formation, poorer mixographic parameters, and lower milling quality (Zhang-Biehn et al., 2021). In hexaploid wheat, *Glu-*1Ay gene is silenced in most cases while it is believed that putting active 1Ay genes in use could be help improve the quality of flour (D. Wang et al., 2018). Moreover, the expression of 1Ay subunits results in improvement of protein and gluten content, increasing dough mixing properties, thus better dough and bread quality (Roy et al., 2020; D. Wang et al., 2018). Hence, in order to better the quality of wheat flour, active *Glu-*1Ay allele could be taken into account in breeding. To support this, Cao et al. (2021) revealed an increase in the overall grain protein content as well as bread making quality through introgression of 1Ay21∗ into commercial wheat cultivars (Cao et al., 2021). Research has demonstrated a positive relationship between inter-chain disulfide bond content and the characteristics of dough (Liu et al., 2016a). The disulfide bond could be formed via various pathways, including: (1) between the cysteines in an y-type HMW-GS N-terminal domain and a residue with equivalent characteristics in another y-type HMW-GS, connected in parallel; (2) between the cysteine of an y-type HMW-GS repetitive central domain and that in LMW-GS; (3) between the additional cysteine of 1Dx5 and that of an x-type HMW-GS C-terminal domain (Lutz et al., 2012; D.-W. Wang et al., 2017). In this regard, Wang et al. (2016) demonstrated that adding 1Dx5-N significantly raised gluten network formation via hydrophobic interactions and disulfide bonds cross-linking; this contributes to further improvement in dough quality (Yaping Wang et al., 2016). Furthermore, the proportion of gluten's secondary structure in three near-isogenic lines of wheat majorly originated in various compositions of HMW-GS, which are encoded by *Glu-A1* and *Glu-D1* loci. As a result, in gluten, β-sheets content is significantly associated with the rheological features of dough (Yiwen Li et al., 2015). Compared with normal Bx7 subunits, in the secondary structure of gluten, it was also found that Bx7^OE^ (overexpression of Bx7 subunit caused by gene duplication) subunits of a near-isogenic line of wheat, resulted in augmented β-sheets content. Improved rheological features of dough were also attributed to these subunits (Yi Li et al., 2020). Further β-turns might be generated by either a longer repetitive domain or a higher number of repeat units of HMW-GSs, resulting in higher elasticity potential of polymers; these changes are highly conducive to improving dough, thereby bread making quality (Yi Li et al., 2020; D. Wang et al., 2018). Moreover, another report has shown that dough quality could be positively affected by HMW-GSs’ α-helixes content (Yan Wang et al., 2021). According to a secondary structure prediction in a previous work, in comparison to hexaploid wheat subunits, there is smaller α-helix quantity in certain subunits belonging to a cultivar with low-quality flour (P. Zhang et al., 2016). In contrast, another paper has shown that α-helix content is negatively related to the quality of dough. Li et al. (2023) showed an increase in protein polymerization, a higher amount of glutenin in gluten protein of wheat, faster accumulation of unextractable polymeric protein throughout the development stage of grain, as well as gluten network's denser microstructure over dough preparation by adding *Psathyrostachys huashanica* HMW glutenin subunits (P. Zhang et al., 2016) (see Table 2).

**Table 1:** Allele specific primers of HMW-GS and LMW-GS genes.

HMW-GS genes	Primer sequences ($\overset{´}{5}$-$\overset{´}{3}$)	LMW-GS genes	Primer sequences ($\overset{´}{5}$-$\overset{´}{3}$)
Ax Null	ACGTTCCCCTACAGGTACTA	Glu-D3	CACCAACAGCAACCGA
	TATCACTGGCTAGCCGACAA	Glu-D3	CAAGATAGATGGCTGAACAT
Ax2*	ATGACTAAGCGGTTGGTTCTT	Glu-B3	TCAAAACCAAGCAACACTAT
	ACCTTGCTCCCCTTGTCTTT	Glu-B3	GCTGCTGAGGTTGGTTC
By8	TTAGCGCTAAGTGCCGTCT	Glu-B3	CATCACAAGCACAAGCATCAA
	TTGTCCTATTTGCTGCCCTT	Glu-B3	ACTAGAGATCTTTCCTTATTAG
Bx6, Bx7, Bx17	CGCAACAGCCAGGACAATT	Glu-D3	GCTAGTGCAACCTAACGCAT
	AGAGTTCTATCACTGCCTGGT	Glu-D3	ACGGCACATCGTTGGTA
Dx5	GCCTAGCAACCTTCACAATC	Glu-D3	AAGATCATCACAGGCACAATC
	GAAACCTGCTGCGGACAAG	Glu-D3	CTGCTGACCCAATTGTTGTAG
Dy10, Dy12	GTTGGCCGGTCGGCTGCCATG	Glu-D3	TGCAACCTACCACAATGTCC
	TGGAGAAGTTGGATAGTACC	Glu-D3	GGGTTGGTAGACACCTTGAA
		Glu-D3	TAATTCATTTCAGATGGAGC
		Glu-D3	GGGATTTGTTGTTGCACC
		Glu-A3	CGTCTTTGCCCTCCTCGCTC
		Glu-A3	TTGGGGCTGTTGTTGCTGATA

**Table 2:** The effect of gluten genes on breadmaking quality.

Allele	Locus	Effect on bread making	Reference
*Ax1*	Glu-A1	Ris in dough mixing time, larger bread loaves	(D. Wang et al., 2018)
Ax2*	Glu-A1	Strength in dough, improve bread quality	
*Ax-Null*	Glu-A1	Poor baking quality	Hernández-Estrada et al. (2017)
*Bx7*	Glu-B1a	Improve bread making quality	Espí et al. (2012)
*Bx7* + *By8*	Glu-B1b	Improve bread making quality	
*Bx7* + *By9*	Glu-B1c	Improve bread making quality	
Bx7* + *By8*	GluB1u	Improve bread making quality	(G. Chen et al., 2019b; Yi Li et al., 2020)
Bx7* + By8*	Glu-B1ak	Improve bread making quality	
*Bx7OE + By8*	GluB1al	Stronger dough rheological properties	
1Bx6 + 1By8	Glu-B1d	Poor baking quality	(Cho et al., 2018; Hernández-Estrada et al., 2017; Kaur et al., 2014; Yiwen Li et al., 2015; Liu et al., 2016b; D. Wang et al., 2018)
1Bx17 + 1By18	Glu-B1i	Increase elastic moduli	
Bx13 + By16	Glu-B1f	Good extensibility, higher bread loaf volume	(P. Jiang et al., 2019)
*Bx7* + *By9*	Glu-B1	Raise the consistency, extensibility, viscosity, and elasticity of dough	Nucia et al. (2019)
1Bx20	Glu-B1	Reduce wheat dough strength	Gao et al. (2018b)
*Dx2* + *Dy12*	Glu-D1a	Poor baking quality	(Yelun Zhang et al., 2009)
*Dx5* + *Dy10*	Glu-D1d	Increase in dough's consistency, elasticity, viscosity, and extensibility	(Anderson and Bekes, 2011; Barak et al., 2013b; Hernández et al., 2012; Liang et al., 2010; Morris, 2021; Sissons et al., 2014)
*1Dy12.6, 1Dy12.7*	Glu-D1	Strengthening gluten polymer interactions	Peng et al. (2015)
Subunit 5	Glu-A3b	Positively affects the sedimentation volume of Zeleny gluten strength	Liang et al. (2010)
Subunit 6	Glu-A3a	Increase dough strength	Zhen et al. (2014)
LMW-GS	Glu-A3f, Glu-B3b, Glu-B3g, Glu-B3i, Glu-B3a, Glu-B3d, Glu-B3h, Glu-D3a, Glu-D3c	Increase gluten strength	Bonafede et al. (2015)
LMW-GS	Glu-A3e, Glu-B3a, Glu-B3i	Decrease gluten quality	
Bx6+ By11	Glu-A3d	Good extensibility, higher bread loaf volume	(P. Jiang et al., 2019)
Bx20+ By20	Glu-B1e	Pasta quality-improving	Varzakas et al. (2014)
1Bx13 + 1By19	Glu-B1g	pasta quality-improving

#### 3.1.1 Glu- A1

As reported by Wang et al. (2018), stronger dough along with improved baking characteristics were attributed to the HMW glutenin alleles found at *Glu-A1* locus (for example, *Ax1* and Ax2*) (D. Wang et al., 2018). Meanwhile, Ax-Null present at *Glu-A1* locus was associated with poor baking quality (Hernández-Estrada et al., 2017). Therefore, increase in subunit Ax1 contributed to a rise in the mixing time of dough, as well as maximized resistance and mixing tolerance. In terms of size, loaves baked using Ax1 transgenic flours were the same as or larger than loaves made with flour from their non-transformed parent; however, they represented developed crumb grain. According to Vázquez et al. (2012), the 1Ax2* found on Glu-A1 is closely correlated with far more strength in dough and developed quality of baking bread (Vázquez et al., 2012). In addition, Rakszegi et al. (2008) revealed that transgenic lines with high *1Ax1* subunit overexpression led to an over-strong type of dough (Rakszegi et al., 2008).

#### 3.1.2 Glu -B1

Among the three Glu-1 loci, *Glu-B1* has the most diversified variations. Additionally, various *Glu-B1*-encoded HMW-GS compositions have been shown to affect secondary structure proportion, gluten microstructure, and wheat bread making quality (Cho et al., 2018; Gao et al., 2018a).

There are three homologous x-type subunits encoded by the alleles at *Glu-B1*, namely Bx7, Bx7* and Bx7^OE^ (Yi Li et al., 2020). Espí et al. (2012) put subunit Bx7 in HMW-GS in three categories of alleles at locus *Glu-B1*, being *Glu-B1a* (Bx7), *Glu-B1b* (Bx7 + By8), and *Glu-B1c* (Bx7 + By9) (Espí et al., 2012). Given the difference between subunits Bx7* and By8*, and Bx7 and By8 in terms of molecular weight and electrophoretic mobility, over three alleles have been added: *GluB1u* (Bx7* + By8), *Glu-B1ak* (Bx7* + By8*), and *GluB1al* (Bx7^OE^ + By8). Several studies have demonstrated the importance of Bx7 alleles in bread making quality (G. Chen et al., 2019; Yi Li et al., 2020). Thus, Bx7 absence has been associated with negative effects on gluten network's micro-structure, leading to the sponge cake performance (G. Chen et al., 2019). However, different types of wheat containing Bx7^OE^ subunit showed stronger dough rheological properties and improved quality of bread baking in comparison to the types with Bx7 subunit (Cho et al., 2018; Yiwen Li et al., 2015). There are very few varieties of wheat including Bx7^OE^ (Nucia et al., 2019) and breeding further varieties of it, which contain Bx7^OE^ subunit, could be a promising alternative for improving the quality of wheat. Studies have shown that the contents of β-sheets and β-turns would positively relate with dough elasticity while α-helix content has been found to be a negative effect. Accordingly, Bx7^OE^ with augmented dough strength contributes to more β-sheets and β-turns than α-helices (G. Chen et al., 2019; Gao et al., 2018a; Yi Li et al., 2020; Liu et al., 2016a). Growing β-sheet content is associated with strikingly enhanced Bx7 subunit expression, which harbors long repetitive domain with further β-sheets in its secondary structure. As a result, Bx7^OE^ promotes dough rheological properties. In addition to β-sheets content, more free sulfhydryl groups were identified in HMW-Bx7^OE^ in comparison with HMW-Bx7 (Yi Li et al., 2020). According to Delcour et al. (2012), by oxidizing free sulfhydryl groups into inter-molecular disulfide bonds, glutenin macropolymers would be produced during the formation of the dough (Delcour et al., 2012). Yi Li et al. (2020) also showed HMW-Bx7^OE^ gluten surface to be denser, smaller, and deeper than that of HMW-Bx7, leading to higher bread quality. Moreover, Bx7 in combination with other alleles has positive effects on bread making quality (Yi Li et al., 2020).

*The combinations of* Glu-B1 *alleles affect bread making quality through different ways;* 1Bx6 + 1By8 is associated with poor baking quality while higher elastic moduli are attributed to 1Bx17 + 1By18, 1Bx13 + 1By16 and 1Bx7 + 1By8 at Glu-B1. They could result in stronger dough, positively affecting the volume of bread, thereby baking properties (Cho et al., 2018; Hernández-Estrada et al., 2017; Kaur et al., 2014; Yiwen Li et al., 2015; Liu et al., 2016a; D. Wang et al., 2018). In this regard, Nucia et al. (2019) showed that Bx7 + By9 raise the consistency, extensibility, viscosity, and elasticity of dough, improving baking quality (Nucia et al., 2019). León et al. (2009) also reported the following order regarding the positive effect of *Glu-B1* locus-encoded HMW-GSs on the quality of bread making: Bx17 + By18 > Bx14 + By15 > Bx7 +By8 > Bx7+By9 (León et al., 2009). In another study conducted by Gao et al. 2018a, b, 1Bx20 insertion reduced wheat dough strength due to two less cysteines. Out of the four near-isogenic lines, the highest viscoelasticity and β-turns content belonged to NIL 2 with Bx14 + By15 (Gao et al., 2018a). Meanwhile, the highest strength in wheat dough and β-sheet content were observed in NIL 3 with Bx17 + By18 (Gao et al., 2018a). However, Cui et al. (2019) applied 1Sl-encoded high molecular weight glutenin subunits (HMW-GS), 1Slx2.3*, and 1Sly16* from *Aegilops longissima* L., in spring wheat cultivar and concluded that these subunits conveyed better dough rheological properties and higher bread making quality than the 1Bx17 + 1By18 subunits (Cui et al., 2019).

#### 3.1.3 Glu - D1

A substantially important element in bread quality is the D-subgenome locus; the absence of D-genome leads to major differences in the quality of dough in tetraploid pasta wheats (D.-W. Wang et al., 2017). In general, the most influential factor on the characteristics of dough and bread making is known to be *Glu-D1* loci, following which *Glu-B1* and *Glu-A1* could be respectively mentioned (Nucia et al., 2019; Yang et al., 2014; P. Zhang et al., 2016). *Glu-D1a* (SDS-PAGE allele designation 2 + 12) and *Glu-D1d* (5 + 10) are two alleles at *Glu-D1*, frequently seen in bread wheat. *Glu-D1d* (5 + 10) results in further improvement in the quality of bread making (Yelun Zhang et al., 2009). A considerable body of evidence has additionally indicated the significant effects of each of *Glu-D1* and *Glu-B1* on dough features, independent of each other, while *Glu-A1* loci are dependent on other *Glu-1* subunits in order to affect dough (P. Jiang et al., 2019). In general, the cultivars including closely linked alleles *Glu-D1-1b* and *Glu-D1-2b*, respectively encoding subunits Dx5 and Dy10, have stronger doughs compared to the cultivars with *Glu-D1*-*1a* and *Glu-D1*-*2a* that encode Dx2 and Dy12 subunits. Various studies have revealed that 1Dx5 + 1Dy10 alleles determine the level of increase in dough's consistency, elasticity, viscosity, and extensibility; these features play a pivotal role in the quality of baking and appropriate volume of loaf (Anderson and Bekes, 2011; Barak et al., 2013; Hernández et al., 2012; Liang et al., 2010; Morris, 2021). Subunit Dx5 has an additional cysteine for an inter-chain crosslink compared to other x-type subunits (Rhazi et al., 2014). of the absence of Dx5+Dy10 has a negative effect on gluten strength and the quality of bread; that said, increasing Dx5 or Dy10 subunits results in stronger dough (P. Jiang et al., 2019; Naeem et al., 2012) (Fig. 4). Research has also demonstrated that overexpressed Dx5 raises the mixing time while diminishing the peak resistance; this might lead to over strong dough that is not suitable for making bread (León et al., 2009; Yan Wang et al., 2021). Furthermore, in transgenic wheat, Dy10 expression enhances the time of dough development and mixing tolerance whereas its absence in the mutant probably contributes to the restructuring of the inherent network of gluten, thereby reduced strength of dough (León et al., 2009; Yan Wang et al., 2021). Comparing Dx5 to Dy10 subunit, the latter has more cysteine residues which create inter-molecular disulfide bonds in the course of dough development. This enables glutenin polymers extensive cross-linking. Anderson and Bekes (2011) demonstrated Dx5 and Dy10 subunits to synergistically interact via their N-terminal domains, where a direct relationship exists between dough features and the repetitive domain length (Anderson and Bekes, 2011). As reported by Naeem et al. (2012), the polymerization of HMW-GSs, which are related to dough strength (Dx5 + Dy10 for example), is earlier (Naeem et al., 2012). Compared with the polymerization of HMW-GSs related to dough weakness (*1Dx2* + *1Dy12* for example). In biscuit production, purified Dy10 incorporation into wheat flour reduced the biscuit area, enhanced its thickness, and diminished the spread ratio; this suggests that biscuit quality is negatively correlated with the Dy10 content (H. Chen et al., 2021). Not only Dy10 and Dx5, but also Dy3, are known as effective subunits of HMW-GS in terms of wheat quality (Aghagholizadeh et al., 2017). Wang et al. (2018), Hernández-Estrada et al. (2017), and Kiszonas and Morris (2018) introduced 1Dx2 + 1Dy12 as the alleles associated with poor baking quality, being more suitable for soft wheat/pastry end uses (Hernández-Estrada et al., 2017; Kiszonas and Morris, 2018; D. Wang et al., 2018). In contrast with the studies suggesting the positive effects of subunit pair Dx5 + Dy10 on bread making, Mohamed et al. (2022) reported this subunit pair from *Triticum tauschii* as poor genome concerning its effect on dough strength (Mohamed et al., 2022). They suggested that decreased dough strength values in subunit pair Dx5+Dy10 could be due to the lack of extra cysteine in *T.* tauschii-derived 1Dx5 as observed in previous works. Moreover, Mohamed et al. (2022) showed the lines carrying 1Dx2 + 1Dy12 derived from *T. tauschii* to result in stronger dough in spite of being frequently attributed to lower dough strength (Mohamed et al., 2022). This finding may refer to the high amount of total HMW-GS at the Glu-D1 locus in this subunit pair, as noted previously. *T. tauschii* is therefore known as a reservoir for unique Glu-D1 alleles (Dx2 + Dy12 and Dx5 + Dy10), providing the genomic resource for utilizing new alleles in order to improve end-use quality in programs designed for wheat breeding (Delorean et al., 2021). Peng et al. (2015) introduced 1Dy12.6 and 1Dy12.7 subunits and revealed that they are capable of strengthening gluten polymer interactions, which makes them vital genetic resources for ameliorating the quality of wheat (Peng et al., 2015).

#### 3.1.4 LMW-GS

HMW-GS role in bread making quality is further studied whereas LMW-GS is also of particular significance in the creation of large polymers. Approximately 50% of gluten proteins is constituted by LMW-GS which is conducive to technological quality by 30%. Despite the low number of HMW-GSs, a multigene family encodes LMW-GSs; this family is at the *Glu-A3*, *Glu-B3*, and *Glu-D3* loci respectively on the short arms of chromosomes 1A, 1B, and 1D (Tables 1 and 2; Fig. 3B) (Rasheed et al., 2014). Glu3 loci are strongly linked with gliadin encoding sites and of great importance in the quality of bread making (Goldasteh et al., 2019; Lee et al., 2017). According to N-terminal end, are three kinds of LMW-GS, namely LMW-i, LMW-m, and LMW-s. LMW-i is correlated with isoleucine residue while LMW-m is associated with methionine residue and LMW-s with serine residue. Nearly similar peptide sequences were observed in LMW-i and LMW-s, with the latter being marginally more hydrophobic due to the presence of serine instead of the isoleucine in LMW-i. LMW glutenin alleles along with gliadins are significantly correlated with dough extensibility (Patil et al., 2011). Moreover, LMW-GS is capable of creating inter-molecular disulfide bonds, either with each other or with HMW-GS. LMW-GS is also known as a pivotal element for gluten polymer to be created (Patil et al., 2011). The majority of LMW-GSs include eight cysteine residues, with three types of subunits varying in position. In gluten macropolymer, these cysteine residues are essential in the creation of intra- and inter-molecular disulfide bonds (Beom et al., 2018). Compared with durum wheat, the abundance of Glu-D3-encoded LMW-GS may result in the viscoelasticity of gluten in common wheat (Patil et al., 2011). Additionally, concerning LMW-GS, it was reported that Glu-A3b (subunit 5) positively affects the sedimentation volume of Zeleny as well as gluten strength (Liang et al., 2010). Wang et al. (2016) showed that Glu-B3h (an individual allele at Glu-3 loci) deletion resulted in an obvious reduction in bread mixing properties, dough strength, and loaf volume (Yaping Wang et al., 2016). Moreover, (Zhen et al., 2014) revealed that deleting Glu-A3a (subunit 6) remarkably decreased the strength of dough, thereby the quality of bread making. (Bonafede et al., 2015) attributed Glu-A3f, Glu-B3b, Glu-B3g, and Glu-B3i alleles to the highest values in the parameters related to gluten strength. Meanwhile, according to them, Glu-A3e, Glu-B3a, and Glu-B3i are invariably related to low-quality values and weak gluten. In accordance with other papers, Franaszek and Salmanowicz (2021) revealed that different types of wheat with Glu-3 loci scheme (Glu-A3b, Glu-A3f at the Glu-A3 locus; Glu-B3a, Glu-B3b, Glu-B3d, Glu-B3h at the Glu-B3 locus; Glu-D3a, Glu-D3c at the Glu-D3 locus) were indicative of the most important quality-improving factors (Franaszek and Salmanowicz, 2021).

## 4 Genetic characteristics of durum wheat

The variety in *Triticum durum* and *Triticum aestivum* attributes is on account of different genetic and physiochemical characteristics. Compared to bread wheat (hexaploid (AABBDD)) grains, those of durum wheat (tetraploid (AABB)) are known to be more vitreous, larger, and harder. The absence of D genome in *Triticum durum* is responsible for the reduction in its baking performance (Zarroug et al., 2015). The quality of pasta cooking closely depends on the protein content of flour and gluten's strength (Sissons et al., 2014). The allelic forms of HMW-GSs and LMW are considered as major determinants of gluten strength.

Regarding the genetic aspect, in tetraploid durum wheat, there are four HMW-GSs on the long arm of homologous chromosome 1A and 1B (Glu-A1 and Glu-B1) (Janni et al., 2018; Yi Li et al., 2020). There is a pair of closely linked genes in every locus, one of which encodes an x-type glutenin subunit while the other one encodes an y-type. By silencing the genes, there are normally only one to three accumulated GSs in the endosperm (Janni et al., 2018). Both *Glu-B1d (6 + 8)* and *Glu-B1h (14 + 15)* have been related to the parameters important in dough quality, resistance breakdown value, and SDS sedimentation value. Nonetheless, Glu-B1d was found to be also advantageous to improve the quality of biscuit production. Similarly, the high frequency of *Glu-B1b (7 + 8)* (23/152 entries) might originate in its relationship with higher gluten strength and pasta quality (Nazco et al., 2014). Sissons et al. (2014) documented the following order of ranking for Glu-B1 alleles based on their pasta quality-improving effects: *Glu-B1b (7 + 8) > Glu-B1e (20 + 20) > Glu-B1d (6 + 8)* (Sissons et al., 2014), another ordering adjusted by (Varzakas et al., 2014) in order to take into account less common alleles to *Glu-B1i (17 + 18)* > *Glu-B1g (13 + 19)* > *Glu-B1(7 + 8)* > *Glu-B1a (7)* > *Glu-B1d (6 + 8)* (Sissons, 2008; Varzakas et al., 2014). Moreover, Sissons et al. (2014) demonstrated that adding subunits Dx2 + Dy12 and Dx5 + Dy10, which are usually present at the Glu-D1 locus in bread wheat, resulted in lower cooked firmness in pasta made of these genotypes (Sissons et al., 2014). However, Kiszonas et al. (2021) reported that dough strength increased by introducing Glu-D1 alleles, namely Glu-D1a and Glu-D1d, into durum wheat, which correspond to HMWG subunits Dx2 and Dy12, respectively (Kiszonas et al., 2021). This is confirmed by the significant increase in SDS sedimentation volume and mixograph mixing parameters. Furthermore, Camerlengo et al. (2022) introduced Gli-D1/Glu-D3 and Glu-D1 loci into durum wheat genomes and obtained the maximum values of gluten index (dough strength and extensibility) as well as superior bread making characteristics (Camerlengo et al., 2022). In addition, Glu-B1x and Glu-B1y have a reputation for their mixed effects on the quality of pasta. Their individual loss-of-function mutants (ΔBx6 and ΔBy8, respectively) were also correlated with a significant decline in gluten strength and increased cooking loss compared to the wildtype (Yazhou Zhang et al., 2020).

## 5 Challenges and future work

Ethical, safety, and regulatory considerations surrounding genetic manipulation are of utmost importance. While genetic manipulation can offer potential benefits in improving bread quality and addressing certain limitations, it is crucial to ensure that these modifications are carried out responsibly and with consideration for various factors.

Ethical considerations involve the assessment of the potential impacts and consequences of genetic manipulation. This includes evaluating the potential effects on human health, the environment, and the overall sustainability of agricultural practices. It is essential to consider the potential unintended consequences of genetic manipulation and weigh them against the potential benefits.

Safety considerations are paramount to ensure that any genetic modifications do not pose risks to human health or the environment. Thorough assessments and rigorous testing are necessary to determine the safety of genetically modified organisms (GMOs) before their introduction into the food chain. Regulatory bodies play a crucial role in establishing and enforcing safety regulations and guidelines for the use of genetically modified crops.

Regulatory considerations involve complying with the existing regulations and guidelines set by national and international regulatory bodies. These regulations vary across different countries and regions, and it is essential to adhere to the specific requirements and procedures for the approval and commercialization of genetically modified crops.

Transparency and public engagement are also important aspects of genetic manipulation. Engaging in open and inclusive discussions with stakeholders, including consumers, farmers, and environmental groups, can help address concerns, ensure informed decision-making, and build trust in the process of genetic manipulation.

Looking into the future, the combination of genomic, functional genomics and genome editing studies will speed up the basic and applied research on gluten proteins, thus enabling efficient development of elite wheat varieties with the end-use traits desired by different consumption needs.

## 6 Conclusion

The quality of wheat is a significant determinant in breeding programs as it affects the commercial value of the cultivar and the quality of the end product. In this work, various wheat cultivation alleles and their effects on bread and pasta wheat quality were thoroughly discussed. According to the studies reviewed, silencing or expression of alleles in gliadin (Gli-A1, Gli-B1 and Gli-D1 loci and Gli-A2, Gli-B2 and Gli-D2 loci), alleles in HMW-GS (Glu-A1, Glu-B1 and Glu-D1), and LMW-GS (Glu*-A3*, *Glu-B3* and *Glu-D3*) could lead to either positive or negative effects on dough and bread quality. In breeding programs, *Ax1*, Ax2*, Bx7, Bx7^OE^, Dx5, Dy10, and their combinations are considered for improving bakery formulations with more desirable gluten strength and extensibility in baking industry. Overall, approaching genetic manipulation with caution, considering ethical, safety, and regulatory considerations, is crucial to ensure responsible and sustainable development in the field. In the case of gluten protein genomes, genetic manipulation would help bakery end product improvement.

## Author contributions

Conceptualization, K.P.; E.A.; S.M.B.H.; supervision, E.A.; investigation, K.P.; E.A.; S.M.B.H.; writing—original draft preparation, K.P.; E.A.; S.M.B.H.; writing—review and editing; K.P.; E.A.; S.M.B.H.

All authors have read and agreed to the published version of the manuscript.

## Funding

This research received no external funding.

## Institutional review board statement

Not applicable.

## Informed consent statement

Not applicable.

## Declaration of competing interest

The authors declare that they have no known competing financial interests or personal relationships that could have appeared to influence the work reported in this paper.

## Data Availability

The data that has been used is confidential.
